# The NHS Health Check in England: an evaluation of the first 4 years

**DOI:** 10.1136/bmjopen-2015-008840

**Published:** 2016-01-12

**Authors:** John Robson, Isabel Dostal, Aziz Sheikh, Sandra Eldridge, Vichithranie Madurasinghe, Chris Griffiths, Carol Coupland, Julia Hippisley-Cox

**Affiliations:** 1 Centre for Primary Care and Public Health, Queen Mary University of London, London, UK; 2 Usher Institute of Population Health Sciences and Informatics, The University of Edinburgh, Edinburgh, UK; 3 School of Community Health Sciences, The University of Nottingham, Nottingham, UK

**Keywords:** PRIMARY CARE, Health check, cardiovascular risk prevention, cardiovascular risk

## Abstract

**Objectives:**

To describe implementation of a new national preventive programme to reduce cardiovascular morbidity.

**Design:**

Observational study over 4 years (April 2009—March 2013).

**Setting:**

655 general practices across England from the QResearch database.

**Participants:**

Eligible adults aged 40–74 years including attendees at a National Health Service (NHS) Health Check.

**Intervention:**

NHS Health Check: routine structured cardiovascular check with support for behavioural change and in those at highest risk, treatment of risk factors and newly identified comorbidity.

**Results:**

Of 1.68 million people eligible for an NHS Health Check, 214 295 attended in the period 2009–12. Attendance quadrupled as the programme progressed; 5.8% in 2010 to 30.1% in 2012. Attendance was relatively higher among older people, of whom 19.6% of those eligible at age 60–74 years attended and 9.0% at age 40–59 years. Attendance by population groups at higher cardiovascular disease (CVD) risk, such as the more socially disadvantaged 14.9%, was higher than that of the more affluent 12.3%. Among attendees 7844 new cases of hypertension (38/1000 Checks), 1934 new cases of type 2 diabetes (9/1000 Checks) and 807 new cases of chronic kidney disease (4/1000 Checks) were identified. Of the 27 624 people found to be at high CVD risk (20% or more 10-year risk) when attending an NHS Health Check, 19.3% (5325) were newly prescribed statins and 8.8% (2438) were newly prescribed antihypertensive therapy.

**Conclusions:**

NHS Health Check coverage was lower than expected but showed year-on-year improvement. Newly identified comorbidities were an important feature of the NHS Health Checks. Statin treatment at national scale for 1 in 5 attendees at highest CVD risk is likely to have contributed to important reductions in their CVD events.

**Strengths and limitations of this study**

- This is the first national study describing implementation of the new National Health Service (NHS) Health Check programme 2009–2012.
- It is based on a large representative sample of 655 general practices in England with 1.68 million people aged 40–74 years eligible for an NHS Health Check of whom 214 295 attended.
- Of those eligible, 70% had ethnic group recorded and 99% socioeconomic group recorded. In attendees, recording of ethnic group and major risk factors was over 90%.
- Non-attendees were younger, more likely to smoke and recording of cardiovascular risk was less complete.
- There is no information available about attendance for support for behavioural change following general practitioner (GP) referral.

## Introduction

The English National Health Service (NHS) Health Checks programme started in 2009, aiming to reduce cardiovascular disease (CVD) risks and events. Internationally, it is the first of its kind, aiming to provide a routine structured clinical assessment and management for adults aged 40–74 years without pre-existing diabetes or CVD. The NHS Health Check includes review of CVD risks, behavioural change support and treatment of newly identified risk factors or comorbidity through integration with routine clinical provision in general practice. We describe an evaluation of the first 4 years of this national programme.

The NHS Health Check is a 5-year rolling programme which targets one-fifth of the eligible population each year, aiming to invite 3 million people at an annual cost of £165 million.^1–3^ The Department of Health report that 2.4 million NHS Health Checks were undertaken in the 2 years (2011–2012).^4^ Nationally, uptake is reported at around 50% of the eligible target population with considerable variability between provider organisations.^4–6^ The NHS Health Check programme is now supported by NHS England and Public Health England following major changes in the NHS in 2013 when Primary Care Trusts (PCTs) were replaced by Clinical Commissioning Groups (CCGs) and responsibility for commissioning the programme was transferred to the Local Authorities.^7^ ^8^

Stratification of CVD risk for the purposes of therapeutic intervention is a key component of the NHS Health Check. Attendees receive personal advice to support behaviour change and treatment informed by CVD risk stratification. When the programme was introduced, National Institute for Health and Care Excellence (NICE) guidance and the NHS Health Check programme,^9^ ^10^ recommended statin treatment at a 10-year CVD risk of 20% or more and antihypertensive treatment with blood pressure sustained at 140/90 mm Hg or more. Comorbidities, including diabetes and chronic kidney disease (CKD), are identified through blood testing in the high CVD risk group with appropriate management. Familial propensity to premature ischaemic heart disease is also identified.

There is robust trial and observational evidence of benefit from statins and antihypertensives in high-risk people with and without established CVD.^11–16^ In people at higher CVD risk, primary prevention of CVD using multiple risk factor intervention including treatment with statins and antihypertensives has been shown to be of benefit.^17^ However, this has not been demonstrated in entire populations including people at lower CVD risk. For people at lower CVD risk (ie, a 10-year risk of <10%) for whom behavioural change is the main intervention, the most effective prevention strategy remains unclear.^16^

Primary prevention based on assessment of cardiovascular risk is a topic of international interest and debate.^18–20^ The study was commissioned by the Department of Health to provide an early view of implementation of the national programme. This study describes the results from the first 4 years of the NHS Health Check programme, the population coverage and characteristics of those who attended, their recorded CVD risks, new comorbidity and treatment. Available information on non-attendees is also reported.

## Methods

The study plan and this report conform to the STROBE recommendations for observational studies.^21^

QResearch is a large, nationally representative and validated primary care electronic database containing the health records of 13 million patients registered from 655 general practices using the Egton Medical Information System (EMIS) computer system for at least a year.

For the 4 years (1 April 2009 to 31 March 2013), we included in the study all eligible adults aged 40–74 years if they had been registered for at least a year. We excluded people ineligible for an NHS Health Check, defined by the Department of Health as people with pre-existing vascular disease including hypertension, ischaemic heart disease, stroke or transient ischaemic attack, atrial fibrillation, heart failure, peripheral arterial disease, CKD, familial hypercholesterolaemia, diabetes and those already on statins.^22^

Read codes are used to code clinical data in primary care. NHS Health Check attendance was identified by Read codes for CVD risk assessment or NHS Health Check completed. We were unable to distinguish NHS Health Checks conducted in general practice from those conducted by a third party such as a community pharmacy. For people with an NHS Health Check, we used the date of the Check as the index date for analysis. For those without an NHS Check during the study period, we allocated an index date of 1 April in each year. The NHS Health Check is a rolling 5-year programme, and the total eligible population each year was divided by five to estimate the number eligible in any 1 year. Coverage was defined as the number of attendees in the year, as a proportion of one-fifth of the population eligible in that year.

The total eligible population and people who attended an NHS Health Check were described according to sex, age group (40–49, 50–59, 60–74) and ethnic group. Ethnic groups were combined into Office of National Statistics categories: white (British, Irish, other Caucasian); South Asian (Bangladeshi, Indian, Pakistani); black African; black Caribbean; Chinese; other Asian; other (any other recorded ethnic group including mixed ethnic groups) and ethnic group not recorded.^23^

Deprivation was assessed using the Townsend score based on 2001 census-derived measures of overcrowding, car ownership and education available at lower super output area.^24^ This was obtained by linking the individuals’ postcode to lower super output area (approximately 150 households). Townsend score was accessible for 99% of patients. We grouped individuals into fifths of deprivation, with quintile 1 indicating least deprived and quintile 5 most deprived.

Information on smoking status, alcohol intake and risk factor recording was described for attendees and non-attendees. This included the latest information recorded up to and including the date of the NHS Health Check for attendees or the index date for non-attendees. Family history of ischaemic heart disease was coded as positive if a first-degree relative had angina or a heart attack under age 60 years. Information on alcohol consumption was categorised by units consumed per day (non-drinker, <1, 1–2, 3–6, 7–9, >9+) although it was not nationally part of the NHS Health Check during the study period. Information was also extracted on whether a recorded CVD risk score was estimated by either Framingham or QRisk2 using the same time frame as specified above. Where a score was recorded we used it to identify people at high CVD risk, defined as a 10-year CVD risk of 20% or more.

In people who attended a Health Check, information was extracted on medications, new morbidities, risk factor recording and referrals on the date of the check or in the following 12 months. The equivalent information was extracted for non-attendees for the 12 months from their index date. New medication was defined as at least two statin or antihypertensive prescriptions within 12 months. New comorbidities, including diagnosed hypertension, CKD categories 3–5 and diabetes, were included if newly recorded within 12 months of an NHS Health Check. Abnormal measurements were not classified as a diagnosis unless a diagnostic code was recorded. For example, a raised blood pressure was not classified as hypertension unless the diagnostic code for hypertension was recorded.

The data were analysed using STATA V.13 (STATA Corps). We calculated proportions of people who attended by categories of age, sex and ethnic group. We calculated proportions according to levels of smoking status, alcohol intake and risk factors in those who did and did not attend an NHS Health check. We also described CVD risk levels and outcomes in attendees following the NHS Health Check. We did not carry out statistical comparisons of NHS Health Check attendees with non-attendees, as data were incomplete in the latter.

## Results

**Figure 1:**
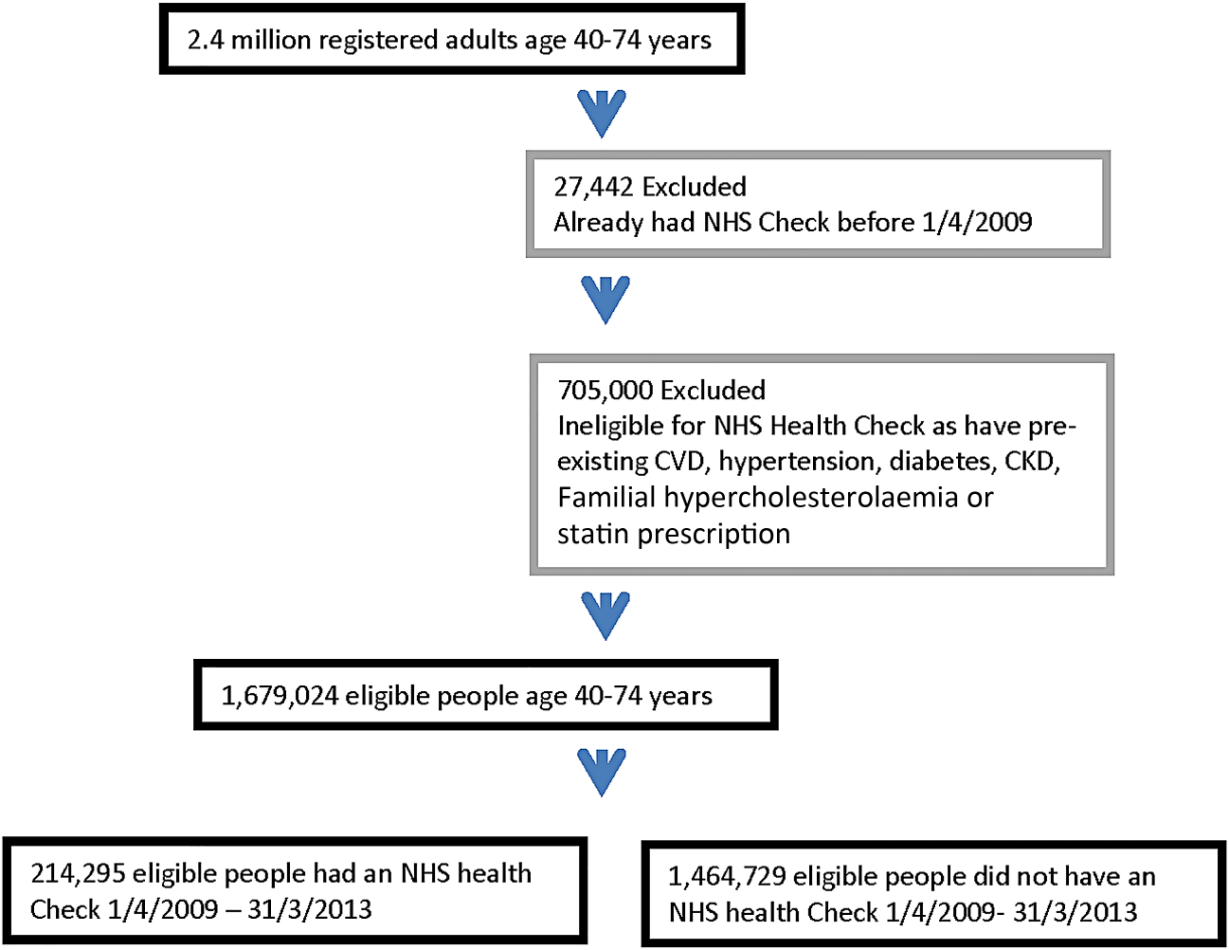
Flow-chart showing inclusion and exclusion of people eligible for an NHS Health Check and attendance. CKD, chronic kidney disease; CVD, cardiovascular disease; NHS, National Health Service.

Over the 4-year study period (1 April 2009–31 March 2013) 1 679 024 people were eligible for an NHS Health Check. Of these, 12.8% (214 295) patients were recorded as having had an NHS Health Check (see figure 1).

One-fifth of the eligible population was considered available for attendance each year.

Table 1 shows coverage by financial year. In 2009/2010, there were a total 1 430 174 people eligible of whom 286 035 (one-fifth) were considered eligible in the year, and of these, 5.8% (16 613/286 035) attended an NHS Health Check. In 2010/11, 14.6% attended; in 2011/12, 24.4% attended; and in 2012/13, 30.1% attended.

**Table 1:** Coverage of National Health Service (NHS) Health Check programme in each year

Financial years	Patients with health check in financial year	20% of the eligible population on 1 April	Percentage of coverage attendance/one-fifth of eligible population
2009/2010	16 613	286 035	5.8
2010/2011	41 832	286 383	14.6
2011/2012	69 978	286 669	24.4
2012/2013	86 042	285 784	30.1

Table 2 shows NHS Health Check coverage for different eligible population subgroups during the entire 4-year study period. Of those eligible aged 60–74 years, 19.6% attended, and at age 40–59 years, 9.0% attended. In the most deprived quintile, 14.9% attended, and in the least deprived quintile, 12.3% attended.

**Table 2:** Characteristics of people aged 40–74 years eligible for a National Health Service (NHS) Health Check between April 2009 and March 2013 and those who attended

	Total eligible people	NHS Health Check attendees	Percentage of total eligible population with NHS Health Check
Total	1 679 024	214 295	12.8
Female	846 797	111 740	13.2
Male	832 227	102 555	12.3
Age band (years)			
40–49	806 199	72 903	9.0
50–59	499 725	68 428	13.7
60–74	373 100	72 964	19.6
Townsend quintile of deprivation			
1 (most affluent)	336 174	41 423	12.3
2	334 996	40 342	12.0
3	335 706	40 897	12.2
4	335 302	41 557	12.4
5 (most deprived)	334 652	49 974	14.9
Townsend not recorded	2194	102	4.7
Ethnicity			
White	1 065 171	185 082	17.4
Indian	16 842	2987	17.7
Pakistani	8472	1362	16.1
Bangladeshi	4925	1460	29.6
Other Asian	13 471	1966	14.6
Caribbean	12 908	2531	19.6
Black African	19 899	3128	15.7
Chinese	6913	1059	15.3
Other	26 045	4059	15.6
Not recorded	504 378	10 661	2.1

Seventy per cent (1 174 646/1 679 024) of the eligible population had ethnic group recorded. There was low attendance, 2.1% among the 30% of the eligible population without ethnicity recorded. Among those with ethnic group recorded, coverage was highest among South Asians (Indian, Pakistani, Bangladeshi, other Asian) of whom 19.2% of the eligible population attended, and black Caribbeans 19.6%, and lowest in black Africans 15.7% and Chinese 15.3%. In white people, 17.4% attended compared with 16.9% in all other recorded ethnic groups.

Table 3 shows information on risk factor recording and CVD risk score recording among attendees and non-attendees prior to or at the NHS Health Check or the equivalent index date for non-attendees. Smoking status was recorded in 99.9% of attendees and 94.5% of non-attendees. Non-attendees were more likely to be smokers. In total, 17.7% of attendees were smokers, and 22.4% of non-attendees were smokers. Alcohol consumption was recorded for 95.9% of attendees and 80.3% for non-attendees. Among those in whom alcohol consumption was recorded, heavy drinking (>9 units/day) was reported by 2.5% of attendees and 2.2% of non-attendees.

**Table 3:** Characteristics of eligible people who did and did not attend for a NHS Health Check recorded prior to or on the date of the NHS Health Check or relevant index date

	NHS Health Check N (%)	No NHS Health Check N (%)
Total	214 295	1 464 729
Smoking status recorded	214 020 (99.9)	1 384 707 (94.5)
Non-smoker	117 968 (55.1)	768 276 (55.5)
Ex-smoker	58 244 (27.2)	306 397 (22.1)
Light smoker (1–9/day)	19 589 (9.2)	167 592 (12.1)
Moderate smoker (10–19/day)	11 052 (5.2)	83 585 (6.0)
Heavy smoker (20+/day)	7167 (3.3)	58 857 (4.3)
Alcohol intake		
Alcohol status recorded	205 506 (95.9)	1 175 900 (80.3)
Non-drinker	53 485 (26.0)	292 289 (24.9)
Trivial <1 units/day	66 780 (32.5)	421 139 (35.8)
Light 1–2 units/day	37 398 (18.2)	205 572 (17.5)
Moderate 3–6 units/day	42 467 (20.7)	227 987 (19.4)
Heavy 7–9 units/day	3235 (1.6)	17 169 (1.5)
Very heavy >9 units/day	1866 (0.9)	8842 (0.8)
Drinker—amount not recorded	275 (0.1)	2902 (0.2)
Risk factor recording		
Body mass index recorded	210 062 (98.0)	1 176 819 (80.3)
Systolic blood pressure recorded	213 690 (99.7)	1 316 926 (89.9)
Cholesterol recorded	195 994 (91.5)	633 548 (43.3)
Cholesterol/HDL recorded	174 345 (81.4)	433 594 (29.6)
Positive family history CHD	46 466 (21.7)	156 604 (10.7)
Mean body mass index (SD)	27.1 4.5)	26.4 (4.6)
Mean cholesterol (SD)	5.5 (1.0)	5.4 (1.0)
Mean cholesterol/HDL (SD)	4 (1.3)	4 (1.2)
Mean SBP (SD)	130.8 (16.9)	128.4 (15.6)
Mean DBP (SD)	79.4 (10.0)	78.6 (9.4)
QRisk2 recorded	171 441 (80.0)	424 523 (29.0)
QRisk2 not recorded	42 854 (20.0)	1 040 206 (71.0)
QRisk2 score		
<5%	47 794 (22.3)	195 253 (13.3)
5–9.99%	43 687 (20.4)	114 706 (7.8)
10–14.99%	32 452 (15.1)	55 306 (3.8)
15–19.99%	22 639 (10.6)	31 356 (2.1)
20+%	24 869 (11.6)	27 902 (1.9)
Framingham score recorded	33 260 (15.5)	76 051 (5.2)
Not recorded	181 035 (84.5)	1 388 679 (94.8)
Framingham score		
<5%	7152 (3.3)	20 532 (1.4)
5–9.99%	10 196 (4.8)	25 898 (1.8)
10–14.99%	6896 (3.2)	15 286 (1.0)
15–19.99%	4283 (2.0)	7787 (0.5)
20+%	4733 (2.2)	6547 (0.4)
20+ QRisk2 or Framingham	27 624 (12.9)	32 481 (2.2)

CHD, coronary heart disease; DBP, diastolic blood pressure; HDL, high-density lipoprotein; NHS, National Health Service; SBP, systolic blood pressure.

CVD risk using QRisk2 was assessed in 80.0% (171 441/214 295) of attendees and in 29.0% (424 523/1 464 729) of non-attendees and versions of Framingham were used in 15.5% (33 260/214 295) of attendees and 5.2% (76 051/1 464 729) of non-attendees.

Of those attendees with QRisk2 scores recorded, 14.5% (24 869/171 441) were at high CVD risk (10-year risk of 20% or more), and 20.7% (4733/33 260) of those with Framingham scores recorded were at high CVD risk. In total, 12.9% (27 624/214 295) of all attendees were recorded at high CVD risk (20% or more 10-year risk) using either QRisk2 or Framingham. Among non-attendees with QRisk2 recorded, 6.6% (27 902/424 523) were at high CVD risk and 8.6% of non-attendees were at high CVD risk (6547/76 051) using Framingham.

Of those attendees with a QRisk2 score recorded, 46.6% (79 960/171 441) had a CVD risk of 10% or more. In the non-attendees with a recorded QRisk2 score, 27.0% (114 564/424 523) were at 10% or more CVD risk.

## New comorbidity

Table 4 records new comorbidity identified at or in the 12 months after the NHS Health Check from 2009 to 2012. This included 7844 new cases of hypertension (1 case per 27 NHS Health Checks), 1934 new cases of diabetes (1 new case for every 110 Checks) and 807 new cases of CKD (1 new case in every 265 Checks).

**Table 4:** Outcomes for people who did and did not have an NHS Health Check (number, %) recorded at NHS Health Check, relevant index date or in 12 months following these dates

	NHS Health Check N (%)	No NHS Health Check N (%)
Total patients	214 295	1 464 729
New diagnoses		
Chronic kidney disease (eGFR <60 mL/min/)	807 (0.4)	2310 (0.2)
Type 2 diabetes	1934 (0.9)	5647 (0.4)
Hypertension	7844 (3.7)	16 184 (1.1)
Risk factor recording		
Body mass index recorded	151 480 (70.7)	144 756 (9.9)
Positive family history of premature CHD recorded	14 760 (6.9)	4720 (0.3)
Blood pressure recorded	140 995 (65.8)	242 928 (16.6)
eGFR recorded	59 021 (27.5)	160 843 (11.0)
Fasting glucose	35 801 (16.7)	78 934 (5.4)
Random glucose	64 439 (30.1)	102 568 (7.0)
Total cholesterol recorded	123 342 (57.6)	137 207 (9.4)
Cholesterol/HDL ratio recorded	118 930 (55.5)	115 011 (7.9)
Smoking status recorded	188 282 (87.9)	410 301 (28.0)
Risk factors identified (as % recorded)		
Fasting glucose≥7 mmol/L	954 (2.7)	2983 (3.7)
Random glucose≥11 mmol/L	421 (0.6)	1291 (1.3)
Raised BP: SBP ≥140mmHg or DBP ≥90mmHg	26 126 (18.5)	52 236 (21.5)
Obesity BMI≥30 kg/m^2^	32 133 (21.2)	39 774 (27.5)
New referrals (as % recorded)		
Current smokers referred to smoking cessation clinic	2571 (6.8)	9944 (3.2)
Weight referrals: BMI ≥30 kg/m^2^	12 430 (38.7)	4441 (11.2)
Exercise referrals: BMI ≥30 kg/m^2^	13 309 (41.4)	4082 (10.3)
Alcohol referrals: >6 units/day	1823 (33.9)	1459 (5.1)
New medication		
2+ prescriptions for statins	10 900 (5.1)	15 086 (1.0)
2+ prescriptions for antihypertensives	8457 (3.9)	26 178 (1.8)

BMI, body mass index; CHD, coronary heart disease; DBP, diastolic blood pressure; eGFR, estimated glomerular filtration rate; HDL, high-density lipoprotein; NHS, National Health Service; SBP, systolic blood pressure.

Recording of new comorbid conditions in the year after an NHS Check was higher in people attending NHS Health Checks than recording in the year after the index date in non-attendees. However, in non-attendees, missing data are a major issue which precludes simple direct comparison between attendees and non-attendees. Risk factors requiring further follow-up were recorded in more than one in five attendees. Raised blood pressure (≥140/90 mm Hg; but not recorded as a diagnosis of hypertension) was identified in 18.5% (26 126/140 995), obesity (body mass index (BMI) ≥30 kg/m^2^) in 15.0% (32 133/151 480) and raised fasting blood sugar (but not recorded as a diagnosis of diabetes) in 2.7% (954/35 801).

In total, 33.9% of heavy and very heavy alcohol drinkers (1823/5376) were referred for further advice, and in people who were obese (BMI ≥30 kg/m^2^), 38.7% (12 430/32 133) received advice on weight reduction and 41.4% (13 309/32 133) on physical activity. In total, 6.8% (2571/37 808) of smokers were referred to dedicated smoking cessation services.

New recurrent prescriptions for statins (two or more prescriptions within 12 months) were provided for 5.1% (10 900) of attendees and new recurrent prescriptions for antihypertensives for 3.9% (8497) of attendees. Equivalently 1 in 20 attendances resulted in recurrent statin prescriptions, and 1 in 25 attendances resulted in recurrent antihypertensive prescriptions.

## Attendees at high CVD risk

Table 5 shows the characteristics of those at high CVD risk (≥20% risk) who attended for an NHS Health Check. In total, 12.9% (27 624/214 295) attendees were recorded at high CVD risk. As expected, those at high CVD risk were older, with 81.0% of high-risk attendees in the 60–74-year age group compared with 34.0% of all attendees. Men comprised 78.3% in the high-risk group and 47.9% among all attendees.

**Table 5:** Characteristics of people at high CVD risk who attended an NHS Health Check 1 April 2009 to 31 March 2013 recorded at or prior to the date of the NHS Health Check

	NHS Health Check risk ≥20% N (%)
Total patients all CVD risks	214 295
Total with CVD risk ≥20%	27 624
Females	5992 (21.7)
Males	21 632 (78.3)
Age band (years)	
40–49	778 (2.8)
50–59	4475 (16.2)
60–74	22 371 (81.0)
Townsend quintile	
1 (most affluent)	5135 (18.6)
2	5356 (19.4)
3	5301 (19.2)
4	5284 (19.1)
5 (most deprived)	6539 (23.7)
Ethnicity	
Ethnicity recorded	26 392 (95.5)
White	25 037 (90.6)
Indian	376 (1.4)
Pakistani	264 (1.0)
Bangladeshi	276 (1.0)
Other Asian	135 (0.5)
Caribbean	89 (0.3)
Black African	27 (0.1)
Chinese	17 (0.1)
Other	171 (0.6)
Not recorded	1232 (4.5)
Smoking status recorded	27 611 (100.0)
Non-smoker	10 517 (38.1)
Ex-smoker	9351 (33.9)
Light smoker (1–9/day)	4024 (14.6)
Moderate smoker (10–19/day)	2199 (8.0)
Heavy smoker (20+/day)	1520 (5.5)
Alcohol intake prior to or at NHS Health Check	
Alcohol status recorded	26 765 (96.9)
Non-drinker	6897 (25.0)
Trivial <1 units/day	7919 (28.7)
Light 1–2 units/day	4684 (17.0)
Moderate 3–6 units/day	6322 (22.9)
Heavy 7–9 units/day	601 (2.2)
Very heavy >9 units/day	304 (1.1)
Drinker: amount not recorded	38 (0.1)
Risk factor recording prior to or at NHS Health Check	
Body mass index recorded	27 243 (98.6)
Systolic blood pressure recorded	27 600 (99.9)
Cholesterol recorded	26 241 (95.0)
Positive family history of premature CHD	9503 (34.4)
Mean body mass index (SD)	27.6 (4.2)

CHD, coronary heart disease; CVD, cardiovascular disease; NHS, National Health Service.

As expected, all other risk factors were more prevalent in those at high CVD risk. Of the high CVD risk attendees, 23.2% (4222/18 203) were obese and 28% (7743/27 611) were smokers. Blood pressure was ≥140/90mm Hg in 30.0% (4772/15 905) at high CVD risk compared with 18.5% (26 126/140 995) in all attendees. Of those NHS Health Check attendees at high CVD risk, 19.3% (5325/27 624) were prescribed recurrent statins and 8.8% (2438/27 624) were prescribed recurrent antihypertensive therapy.

## Referrals to behavioural support

Online supplementary appendix table shows referrals for behavioural interventions in people at high CVD risk. Using data from tables 3 and 4, it can be shown that most referrals took place in people at lower CVD risk (<20% over 10 years). Of those people with behaviourally mediated risk factors recorded—smoking, obesity and high alcohol consumption—who were referred for further support during an NHS Health Check, 80.0% were not in the high CVD risk group. Of the smoking cessation referrals made in smokers, 17.1% (439/2571) were in smokers at high CVD risk and 82.9% were in people at lower risk. Of the dietary referrals made in people with BMI ≥30 kg/m^2^, 13.6% (1691/12 430) were in people at high CVD risk and 86.4% were in people at lower risk. Similarly of the referrals for physical activity, 13.4% (1780/13 309) were in people at high CVD risk and 86.6% were in people at lower risk. Of the total referrals for alcohol reduction support for heavy or very heavy drinkers, 16.5% (300/1823) were in people at high CVD risk and 83.5% were in people at lower risk.

In total, 5.7% (1139/7743) of smokers at high CVD risk were referred to accredited level 2 or 3 smoking cessation services. In people at high CVD risk with BMI ≥30 kg/m^2^, 40.0% (1691/4222) were referred to dietary and 42.2% (1780/4222) to physical activity support services, and 33.1% (300/905) of those at high CVD risk recorded as drinking seven or more units of alcohol per day were referred to alcohol reduction services.

These proportions of people at high CVD risk referred to smoking cessation, dietary, physical activity and alcohol services were very similar to the proportions of all attendees (tables 3 and 4) who were referred, of whom 6.8% (2571/37 808) of smokers were referred to smoking cessation, 38.7% (12 430/32 133) of BMI ≥30 to dietary and 41.4% (13 309/32 133) of BMI ≥30 to physical activity support services, and 35.7% (1823/5101) of heavy drinkers were referred to alcohol reduction services.

## Discussion

This is the first study to describe national results from the NHS Health Check programme. In 2012, the most recent year reported, 30.1% of the eligible population attended. Attendance was more likely over age 65 years 19.6% than in those under 65 years (9.0%) and among those people in the most deprived quintile (14.9%) versus the least deprived (12.3%). In total, 12.9% of all attendees were recorded at high CVD risk (20% or more 10-year risk). There were differences in attendance between ethnic groups, though these could be due to missing data. Attendance in white people was similar to those with non-white ethnic group recorded.

New comorbidity identified in the 4-year period included 7844 new cases of hypertension (1 case per 27 NHS Health Checks), 1934 new cases of diabetes (1 new case for every 110 Checks) and 807 new cases of CKD (1 new case in every 265 Checks). Records of risk factors were more incomplete in non-attendees who had a different CVD risk profile to those who attended. Non-attendees were younger and more likely to be smokers than attendees. Because of differences in the characteristics and recording of risk factors between attendees and non-attendees, we have not made formal statistical comparisons of new morbidity between these groups.

In addition to those people with new comorbidities diagnosed, risk factors such as raised blood pressure, raised blood sugar and obesity requiring further follow-up were recorded in more than one in five of attendees. Most referrals for behavioural interventions took place in people at lower CVD risk. Of those people with behaviourally mediated risk factors recorded—smoking, obesity and alcohol consumption—who were referred for further support during an NHS Health Check, 80% were not in the high CVD risk group. The proportion of people in the high CVD risk group referred because of risk factors was similar to the proportion referred among all attendees.

One in 20 attendances resulted in recurrent statin prescription and 1 in 25 attendances resulted in recurrent antihypertensive prescription. Of those NHS Health Check attendees at high CVD risk, 19.3% were prescribed recurrent statins and 8.8% were prescribed recurrent antihypertensive therapy.

This is a large and nationally representative study including records of social deprivation and ethnicity. Coverage of 30% was lower than expected, though attendance quadrupled during the course of the study reflecting the early phase of implementation. There was no evidence that older people, or those in the more deprived quintile were less likely to attend than other groups. South Asians who have higher CVD risks were more likely to attend than other ethnic groups. Though missing data might account for this, similar differences have been found in other studies.^25^ Currently attendance at NHS Health Checks is reported as uptake in response to invitation rather than coverage, defined as attendance as a proportion of the eligible population as reported in this paper.^4^

The NHS Health Check programme is an example of systematic implementation at national scale, of a stratified approach to advice and effective treatment in people at increased CVD risk. QRisk2 was used in 80% of NHS Checks reported in this study and is fully integrated with the EMIS computer systems used by general practitioners in this study and has since been recommended as the risk algorithm of choice in the 2014 NICE guidance;^26^ an example of successful translation of clinical decision support at scale.^27^ ^28^ This algorithm is fully integrated with the electronic primary care record, a key enabling factor for implementation.^29^

## Limitations of the study

There is no nationally available data on the extent of provision of the NHS Health Check outside of general practices. However, these were likely to represent less than 10% of NHS Health Checks undertaken, as in most PCTs the NHS Health Check was conducted almost entirely in general practice with limited use of community programmes targeting hard-to-reach groups or, with the exception of a few areas, community pharmacies. Completeness of NHS Health Checks was not ascertained, but taking measurement of cholesterol recording of 91.5% as a proxy measure, risk ascertainment was generally well conducted.

Of the people referred with behavioural risk factors, 80% were at lower CVD risk, which indicates the wide distribution of risk factors and the potential for behaviour change if programmes can be shown to be effective. Like other recent studies, referral rates were generally low,^30^ and little is known about attendance at, or quality of behavioural programmes even for those at higher CVD risk. Lack of consistent coding of referrals in earlier years of the NHS Health Check programme and the availability of local services for behavioural change may have contributed to low referral rates. The impact of the NHS Health Check programme on people at lower risk is unknown and further research is required.^31^

The study is descriptive and was not designed to determine variability between practices or CCGs. The study has not assessed changes in risk factors or cardiovascular events between comparable groups. These comparisons are difficult in a non-randomised study, especially if one group is at higher risk than the other or information is incomplete. For these reasons, we have not directly compared attendees with non-attendees. Communication of results and patient behaviours following NHS Health Checks remains an under-researched area.^32–34^

## Implications for practice

A number of local studies suggest that the programme has been better implemented in some areas with coverage of 80% and statin prescription of up to 50% in high-risk individuals in some CCGs.^25^ ^35^ Nationally, uptake in 2011–2012 was reported as 45%, with high levels of variability and better uptake in more deprived areas.^6^ ^5^ There is limited evidence of effectiveness^35^ or comorbidities identified^36^ and statin uptake in those at high risk was reported to be between 20% and 50%,^5^ ^25^ which accords with national surveys of 32%.^37^

Despite a statin treatment rate of only 19% in high-risk attendees in this study, this is likely to have had an important impact on CVD events in those treated. Assuming that 1.2 million people attended a NHS Check each year in the 5 years since 2010, of whom 10% (120 000) were at high CVD risk averaging 2.5% per year, and 19.3% (23 160) of these people were treated with statins over this period and 8.8% (10 560) were treated with antihypertensives; if each treatment reduces cardiovascular risk by 20%, it is estimated that 2529 people would avoid a major CVD event over a 5-year period.^11^ ^12^ Higher uptake in recent years and additional treatment in people at both high CVD risk and at lower CVD risk make this a conservative estimate and behavioural change will have further impact.^37^ These estimates assume that patients who are prescribed medications take them for a 5-year period and that the impact on outcomes is similar to that described in the trial meta-analyses cited.

The NHS Health Check programme has had a difficult birth. The efficacy of the programme has been challenged,^38–41^ based largely on a review of 16 trials of health checks, of which 12 trials were undertaken more than 20 years ago before 1994,^42–52^ the year in which the landmark 4S trial established statin effectiveness.^53^ This means that 12 out of 16 of the reported studies were conducted before statins or modern antihypertensive drugs were used. Of the trials since 1994, only one^52^ specifically recommended drug treatment for CVD risk, the other three offering advice but no drug treatment.^54–56^ The results of the Inter99 study of intensive lifestyle counselling published subsequent to this review^57^ showed no reduction in CVD. Despite high-quality review of trial evidence showing net benefit,^11^ statins have continued to received considerable adverse publicity^58^ ^59^ which has been refuted.^60^

There have also been organisational factors that have impacted on implementation of the programme. Major organisational change in the NHS in the context of financial austerity^61^ ^62^ led to one-third of staff leaving many PCTs in the transition to CCGs in April 2013^63^ and commissioning responsibility for NHS Checks passed to Local Authorities. It is perhaps not surprising that in 2013, 27/151 PCTs nationally offered NHS Health Checks to fewer than 10% of eligible individuals and uptake could be substantially improved.^64^ The most efficient means to deliver this programme including delivery through pharmacies and likely economic impact, remain subjects for further research and debate. A range of infrastructural issues and new research are currently being addressed by Public Health England.^65^ ^66^

This study indicates limited though improving success in the early years of a major new national preventive programme. Coverage of 30% and statin treatment of 19% of attendees at high CVD risk leave considerable room for improvement. New comorbidity and abnormal risk factors were frequently identified in people who attended an NHS Health Check. The majority of referrals for abnormal risk factors were among people at lower CVD risk. This modest start to a major new programme at scale is likely to have made an important impact on CVD events in people who have been treated with statins and antihypertensives or who improved adverse risk factors.
